# Antibiotic resistance genes and molecular typing of *Streptococcus agalactiae* isolated from pregnant women

**DOI:** 10.1186/s12884-023-05380-4

**Published:** 2023-01-19

**Authors:** Mona Zakerifar, Hami Kaboosi, Hamid Reza Goli, Zahra Rahmani, Fatemeh Peyravii Ghadikolaii

**Affiliations:** 1 Department of Microbiology, Ayatollah Amoli Branch, Islamic Azad University, Amol, Iran; 2 Molecular and Cell Biology Research Centre, Faculty of Medicine, Mazandaran University of Medical Sciences, Sari, Iran; 3 Department of Medical Microbiology and Virology, Faculty of Medicine, Mazandaran University of Medical Sciences, Sari, Iran; 4 Department of Obstetrics and Gynecology, Faculty of Medicine, Mazandaran University of Medical Sciences, Sari, Iran; 5 Department of Biology, Qaemshahr Branch, Islamic Azad University, Qaemshahr, Iran

**Keywords:** *Streptococcus agalactiae*, Pregnant women, Antibiotic resistance genes, Molecular typing

## Abstract

**Background:**

The antibiotic resistance of genital tract colonizing *Streptococcus agalactiae* in pregnant women is increasing. We aimed to determine the antibiotic resistance genes of different clonal types of this bacterium in pregnant women.

**Methods:**

Four hundred twenty non-repeated vaginal and rectal specimens were collected from pregnant women and were transferred to the laboratory using Todd Hewitt Broth. The samples were cultured on a selective medium, and the grown bacteria were identified by standard microbiological and biochemical tests. Antimicrobial resistance pattern and inducible clindamycin resistance of the isolates were determined using the disk agar diffusion method. The genomic DNAs of *S. agalactiae* strains were extracted using an extraction kit, and the antibiotic resistance genes and RAPD types were detected using the PCR method.

**Results:**

The average age of the participants was 30.74 ± 5.25 years. There was a significant relationship between the weeks of pregnancy and the number of positive bacterial cultures (*P*-*value* < 0.05). Moreover, 31 pregnant women had a history of abortion, and 18 had a history of membrane rupture. Among 420 specimens, 106 *S. agalactiae* isolates were detected. The highest antibiotic resistance rate was found against tetracycline (94.33%), and all isolates were susceptible to linezolid. Moreover, 15, 15, 42, and 7 isolates showed an iMLS_B_, M-, cMLS_B_, and L-phenotype. The *ermB* was the most prevalent resistance gene in the present study, while 38 (35.84%), 8 (7.54%), 79 (74.52%), 37 (34.9%), and 20 (18.86%) isolates were contained the *ermTR*, *mefA/E*, *tetM*, *tetO*, and *aphA3* gene, respectively.

**Conclusions:**

The high-level antibiotic resistance and prevalence of resistance genes may be due to the arbitrarily use, livestock industry consumption, and the preventive use of antibiotics in pregnant women. Thus, the need to re-considering this problem seems to be necessary.

**Supplementary Information:**

The online version contains supplementary material available at 10.1186/s12884-023-05380-4.

## Background

*Streptococcus agalactiae* (Group B Streptococcus (GBS)) is a Gram-positive coccus colonizing healthy adults that is part of the normal flora of their gastrointestinal and genital tracts [1]. This organism remained unknown until the late 1960s, and then it was noticed in America and Europe as an infectious agent in infants and their mothers [2]. This bacterium causes some problems, including skin and soft tissue infections, sepsis, meningitis, pneumonia, and endocarditis. These infections are more common in infants, pregnant women, and people with underlying diseases, such as diabetes, neurological disorders, cancers, and liver cirrhosis [3, 4]. The vaginal environment provides favorable conditions for the growth and reproduction of *S. agalactiae*. However, 70–80% of colonized mothers may vertically transmit this bacterium to their babies [5]. GBS is responsible for Early-Onset Diseases (EOD) and Late-Onset Diseases (LOD) in infants [6]. The most significant risk factors for GBS infection include the start of childbirth before the 37th week of pregnancy, membrane rupture at least 18 hours before delivery, the presence of a fever higher than 38 °C during delivery, history of invasive disease caused by GBS in a previous baby, and history of urinary tract infection caused by GBS in the current pregnancy [6].

Penicillin and ampicillin are the drugs of choice for treatment of infections caused by *S. agalactiae* [7]. However, clindamycin and erythromycin are used as alternative drugs for patients who are allergic to beta-lactams. Also, the first-generation cephalosporins and vancomycin are alternative antibiotics against beta-lactam-resistant GBS [7]. Today, resistance to penicillin, erythromycin, and clindamycin in *S. agalactiae* isolates has caused concern about the use of these antibiotics [7]. A serious concern is emerging the antibiotic resistance among colonizing GBS due to intrapartum antibiotic prophylaxis in Europe, North America, and Asia [8, 9]. Also, high-level resistance to aminoglycosides, as well resistance against tetracycline, ciprofloxacin, lincomycin, chloramphenicol, and ofloxacin is increasing in GBS isolates [10]. Resistance to macrolides, lincosamides, and streptogramin B (MLS_B_) may occur through efflux pump overexpression, ribosomal alteration, and drug inactivation, leading to a cross-resistance [7]. Erythromycin resistance is caused by ribosomal methylation mediated by *erm* genes (*ermB*, *erm A*/*TR*) encoded methyltransferases [7]. Other reason is the increased expression of efflux pumps, which do not change the drug but expel it. Macrolide efflux pumps (Mef) are encoded by *mefA/E* genes causing macrolide resistance in group B streptococci [11]. Also, lincosamide resistance in GBS is mediated by the production of a nucleotidyltransferase encoded by the *lnu* gene. This enzyme catalyzes the adenylation of the hydroxyl group at the 3-position of lincomycin and clindamycin [12]. There are two MLS_B_ phenotypes, including inducible (iMLS_B_) and constitutive (cMLS_B_). Induced resistance to clindamycin is mediated by activation of mRNA by a methylase in the presence of erythromycin. In this case, resistance to erythromycin leads to the production of a D-shaped no-growth zone around the clindamycin disk [13].

Tetracycline resistance is primarily due to the acquisition of resistance genes associated with mobile genetic elements. However, increased expression of efflux pumps, the presence of ribosomal protective proteins (RPPs), enzymatic inactivation, and a protein with an unknown function called tetU play a role in the emergence of resistance [14]. Tetracycline resistance in GBS is attributed to the TetK and TetL efflux proteins, as well the TetM and TetO RPPs [14]. Also, GBS inherently has low-level resistance to gentamicin. This is due to the low permeability of cell wall against large molecules of aminoglycosides. However, high-level resistance to gentamicin has been observed in some GBS isolates. This resistance is due to the bifunctional aminoglycoside inactivating enzyme (AAC(6′)-APH(2′′)) [15]. This enzyme is predominant in enterococci and staphylococci [15]. On the other hand, *aph-A3* gene encodes an aminoglycoside modifying enzyme, causing aminoglycoside resistance in *S. agalactiae* [16]. Both genes are transposons born with significant distribution among Gram-positive bacteria [15, 16]. Due to the importance of screening pregnant women in terms of colonization with *S. agalactiae* and the antibiotic resistance pattern of the isolates, we aimed to determine the prevalence of different clonal types of this bacterium in pregnant women, along with the antibiotic resistance pattern and the antibiotic resistance genes of the isolates.

## Methods

### Participants and sample size

The study populations were all strains of *S. agalactiae* collected from vaginal and rectal samples of pregnant women from March to September 2021. The participants were referred to Shafa and Nime Shaban hospitals and gynecology clinics in Sari, Mazandaran, Iran. Based on the sample size statistical parameters, 420 clinical isolates were considered for this study. The following formula was used to obtain the sample size, where n = sample size, Z = value of standard normal distribution (Z-statistic) at 95% confidence level (z = 1.96), p = prevalence of colonization with GBS in pregnant women in Iran (*p* = 12.9%) [17], d = maximum error rate = 0.032 [13].

$$n=\frac{z_{\alpha /2}^2\times p\left(1-P\right)}{d^2}$$

### Inclusion criteria

Pregnant women in 35 to 37 weeks of gestation who consented to participate were included in the study. Also, the participants did not use lubricants, sterilizers, vaginal cream, and antibiotics in the last two weeks before sampling. They had no severe diseases, were not hospitalized in the emergency room, and were mentally stable.

### Ethical approval and obtaining the written consent of participants

The demographic information of all participants in this study was recorded and stored in a questionnaire form. Also, necessary explanations regarding voluntary participation were provided to all participants before sampling. Participants filled out the written consent form and were allowed to withdraw from the study whenever they did not want to continue. All data were kept secret, and all methods were performed in accordance with the relevant guidelines and Declaration of Helsinki. Also, our study was approved by the ethics committee of Babol Islamic Azad University, and the code of ethics (IR.IAU.BABOL.REC.1400.058) was assigned to this study.

### Sample collection and transport

According to a previous study, vaginal and rectal swabs provide a high bacterial load for screening GBS [18]. First, a sample was taken from the lower part of (2 cm into) the vagina, periurethral area, and labia of pregnant women by inserting and brushing a cotton-tipped sterile swab by a qualified clinician [19]. The swab was placed in the Todd Hewitt Broth (Sigma, Germany) containing 8 μg/ml of gentamycin, 15 μg/ml of nalidixic acid, and 5% sheep blood [18]. Then, the second sample was taken from the anus (1 cm into the anus and rubbed on the wall of the anal canal) using a cotton-tipped swab by a qualified clinician [19]. The swab was placed in the same Todd Hewitt Broth [18]. The specimens were incubated at 37 °C under 5% CO_2_ for 24 hours, and then were cultured on 5% sheep blood agar plates (Condalab, Spain), containing antibiotics and were incubated.

### Identification of bacteria

The whitish-grey translucent large colonies with a narrow or large zone of β-hemolysis were subjected to the identification tests, including gram staining, catalase, bile esculin, CAMP, susceptibility to bacitracin (0.04 units), and Hippurate hydrolysis [20]. *S. agalactiae* ATCC 12403 and *Streptococcus pyogenes* ATCC 1244 were used as positive and negative control, respectively [19].

### Antimicrobial susceptibility testing

The antimicrobial resistance pattern of GBS isolates was determined using the disk agar diffusion method on 5% sheep blood containing Mueller–Hinton agar (Condalab), according to the Clinical and Laboratory Standards Institute (CLSI) guidelines [21]. The inhibition zone diameters were measured by a calibrated ruler and reported. The antibiotics tested in this study were included penicillin (10 units), vancomycin (30 μg), erythromycin (15 μg), clarithromycin (15 μg), azithromycin (15 μg), clindamycin (2 μg), kanamycin (1000 μg), gentamicin (500 μg), levofloxacin (5 μg), ofloxacin (5 μg), tetracycline (30 μg), chloramphenicol (30 μg), ceftriaxone (30 μg), quinupristin-dalfopristin (15 μg), and linezolid (30 μg) (MAST, UK). High-level amikacin and gentamicin resistant isolates were determined when the kanamycin and gentamicin inhibition zone diameters were < 14 mm and < 17 mm, respectively [22].

### Detection of different macrolide–lincosamide–streptogramin B (MLS_B_) susceptibility phenotypes

The determination of MLS_B_ phenotypes was performed by the double-disk diffusion method on 5% sheep blood Mueller–Hinton agar (Condalab). Briefly, the clindamycin (2 μg) and erythromycin (15 μg) were placed 12 mm apart edge to edge and the plates were incubated for 24 h at 37 °C [21]. Diminishing clindamycin inhibition zone, proximal to erythromycin disk (referred to as D-zone), was detected as inducible MLS_B_ (iMLS_B_) phenotype or inducible clindamycin resistance (ICR) [13, 21]. Also, resistance to both antibiotics with no diminishing clindamycin inhibition zone showed constitutive (cMLS_B_) phenotype [13, 21]. However, resistance to erythromycin but susceptibility to clindamycin with no reducing of inhibition zone around clindamycin disk was considered as M-phenotype (efflux pump overexpression). Also, susceptibility to erythromycin but resistance to clindamycin was determined as L-phenotype [13, 21]. *Staphylococcus aureus* ATCC®BAA-976™ and *Staphylococcus aureus* ATCC®BAA-977™ were used as negative and positive control strains in D-test, respectively [21].

### DNA extraction and polymerase chain reaction

The genomic DNAs of *S. agalactiae* strains were extracted using the SinaPure DNA extraction kit (SinaClon, Iran), based on the manufacturer’s instructions. To confirm the quality of the extracted DNAs, two quantitative and qualitative methods using NanoDrop (Thermo Scientific, USA) and electrophoresis on agarose gel (Wizbiosolutions, South Korea) were used.

Antibiotic resistance genes (*ermB*, *mefA/E*, *ermTR, tetM*, *tetO*, and *aphA-3*) were amplified by PCR using specific primers shown in Table 1. Amplification was done in a final volume of 15 μl, containing 300 ng (1 μl) of the template DNA, five pmol (0.5 μl) of each primer (Bioneer, South Korea), and 7.5 μl of master mix (Ampliqon, Denmark) using a gradient thermocycler (BioRad, USA) in 34 cycles. PCR products were electrophoresed on 1.5% agarose gel (Wizbiosolutions), and were observed after gel staining with Safe stain (SinaClon) in comparison of a 100 bp plus DNA marker (Wizbiosolutions), using a Gel Documentation device (UVITEC Gel Documentation System, Cambridge, UK).

**Table 1:** The primers used for amplification of the antibiotic resistance genes and the PCR conditions

Genes	Primer sequences 5′ to 3′	PCR product size (bp)	Initial denaturation	Denaturation	Annealing	Extension	Final Extension	References
***ermB***	TGGTTTTTGAAAGCCATGCGTCTGA	211	95 °C for 5 min	95 °C for 30 s	60 °C for 30 s	72 °C for 35 s	72 °C for 10 min	[16]
	GGAACATCTGTGGTATGGCGGGTAAGTT							
***MefA/E***	AGTATCATTAATCACTAGTGC	346	95 °C for 5 min	95 °C for 30 s	50 °C for 30 s	72 °C for 35 s	72 °C for 10 min	[16]
	TTCTTCTGGTACTAAAAGTGG							
***ermTR***	GAAGTTTAGCTTTCCTAA	395	95 °C for 5 min	95 °C for 30 s	50 °C for 30 s	72 °C for 35 s	72 °C for 10 min	[16]
	GCTTCAGCACCTGTCTTAATTGAT							
***tetO***	AACTTAGGCATTCTGGCTCAC	515	95 °C for 5 min	95 °C for 30 s	55 °C for 30 s	72 °C for 35 s	72 °C for 10 min	[16]
	TCCCACTGTTCCATATCGTCA							
***tetM***	AGTTTTAGCTCATGTTGATG	1826	95 °C for 5 min	95 °C for 30 s	51 °C for 30 s	72 °C for 35 s	72 °C for 10 min	[16]
	TCCGACTATTTGGACGACGG							
***aphA-3***	AGCTGCCTGTTCCAAAGGTCCTGC	305	95 °C for 5 min	95 °C for 30 s	51 °C for 30 s	72 °C for 35 s	72 °C for 10 min	[16]
	CAGCTCGCGCGGATCTTTAAATGG							

### Typing of *Streptococcus agalactiae* isolates by RAPD-PCR

The vaginal and rectal GBS isolates were genotyped using RAPD-PCR test by a previously used primer (OPS11) with the sequence of 5**′-**AGTCGGGTGG-3**′** [23]. The PCR reaction was performed in a final volume of 15 μl, containing 7.5 μl of master mix (Ampliqon), 1.5 μl (15 pmol) of primer (Bioneer), 4 μl of sterile distilled water, and 2 μl (6 ng) of template DNA. The PCR condition was as follows: An initial denaturation at 94 °C for 5 min and 35 cycles, including a denaturation at 94 °C for 30 s, annealing at 30 °C for 45 s, and an extension at 72 °C for 2 min, followed by a final extension at 72 °C for 10 min. Finally, we electrophoresed the products on a 1.5% agarose gel (Wizbiosolutions) along with a 100 bp plus DNA marker (Wizbiosolutions), and visualized the PCR product using a Gel Documentation device (UVITEC Gel Documentation System). The Dice algorithm was used for the cluster analysis of the isolates, and a UPGMA type dendrogram was drawn. Isolates were defined as the same RAPD clonal type if the Dice coefficient was ≥80%. The different genotypes were assigned based on the number and weight of DNA fragments.

### Statistical analysis

All the results of this study were analyzed using Statistical Package for the Social Sciences (SPSS) software version 22, and a comparison of the data was performed by Chi-square or Fisher’s exact test. The *P*-*value* < 0.05 was considered statistically significant. Data were expressed as count and percent for categorical variables, however, mean and standard deviation (SD) were used to express the quantitative variables. The comparison of two categorical variables was performed by Chi-square, while when the expected values more than 20% of the cells of a contingency table were below 5, we used the Fisher’s exact test.

## Results

### Demographic data of the participants

A total of 420 vaginal and rectal samples were collected from pregnant women referred to hospitals and gynecology clinics, while 106 (25.23%) isolates of *S. agalactiae* were obtained from these samples. Totally, 71 (16.90) rectal and 95 (22.61%) vaginal specimens showed a positive result for *S. agalactiae* culture. Vaginal and rectal specimens were collected from five centers, including Shafa (*n* = 52) and Nime Shaban (n = 5) hospitals, along with Moghadam (*n* = 43), Royan (n = 4), and Shahhosseini (*n* = 2) gynecology clinics. The age range of the studied participants was from 20 to 41 years. The average age of the subjects was 30.74 ± 5.25 years. Most of the participants in this study were in the age groups of 26–30 years (34.9%) and 31–35 years (30.18%). Most of the women investigated in this study (83%) were housewife. Also, 47 pregnant women (44.33%) had education below diploma, while 59 (55.66%) had bachelors to doctorate education. Moreover, 56 (52.83%) participants were hospitalized, and 50 (47.16%) were outpatients. In this study, 61 (57.54%), 18 (16.98%), 19 (17.92%), 6 (5.66%), and 2 (1.88%) pregnant women were experiencing their first, second, third, fourth, and fifth pregnancy.

Also, 12 (11.32%), 26 (24.52%), and 68 (64.15%) participants were in the 35th, 36th, and 37th week of gestation. However, there was a significant relationship between the weeks of pregnancy and the number of positive bacterial cultures (*P*-*value* < 0.05). Among 71 rectal isolates, 10 (14.08%), 15 (21.12%), and 46 (64.78%) were obtained from women in the 35th, 36th, and 37th week of gestation, respectively (*P*-*value* = 0.043). However, out of 95 vaginal isolates of *S. agalactiae*, 10 (10.52%), 23 (24.21%), and 62 (65.26%) were collected from women in the 35th, 36th, and 37th week of gestation, respectively (P-*value* = 0.049). Moreover, out of 106 pregnant women, 31 (29.24%) had a history of abortion. Of these, 19 (61.29%) had a history of one abortion, while 10 (32.25%) and 2 (6.45%) participants experienced two and four abortions. Also, 18 (16.98%) pregnant women had a history of membrane rupture.

### Antimicrobial resistance pattern of the isolates

The antibiotic resistance pattern of the *S. agalactiae* isolates are shown in Table 2. Among the investigated antibiotics, the highest resistance rates were found against tetracycline (94.33%), ofloxacin (78.3%), levofloxacin (67.92%), erythromycin (67.92%), and quinupristin/dalfopristin (60.37%), respectively. On the other hand, all isolates were susceptible to linezolid, while 92.45, 86.79, 78.3, and 71.69% of the isolates were susceptible to vancomycin, gentamicin, penicillin, and chloramphenicol, respectively. The most resistant GBS isolates were collected from Shafa hospital and Moghadam clinic. Also, there was no statistical significant difference between the inpatients and outpatients in terms of resistance to tested antibiotics. Surprisingly, eight isolates were resistant to vancomycin, while six of them were collected from outpatients. Moreover, among 23 penicillin-resistant isolates, 17 (73.9%) were collected from inpatients, while 6 (26%) were obtained from outpatients (*P* = 0.02).

**Table 2:** The antimicrobial resistance pattern of the *S. agalactiae* isolates in the present study

Antibiotics	No. (%) of isolates with different susceptibility pattern			No. (%) of resistant isolates in terms of centers that isolates were collected					No. (%) of resistant isolates in terms of participants’ condition		No. (%) of resistant isolates in terms of abortion		No. (%) of resistant isolates in terms of amniotic sac rupture	
	Resistant	Intermediate resistant	Susceptible	Shafa (n = 52)	Nime Shaban (*n* = 5)	Moghadam (n = 43)	Royan (*n* = 4)	Shahhosseini (n = 2)	Inpatient (*n* = 56)	Outpatient (*n* = 50)	Yes (*n* = 31)	No (*n* = 75)	Yes (*n* = 18)	No (*n* = 88)
Penicillin	23 (21.69)	0	83 (78.3)	16 (30.7)	2 (40)	5 (11.6)	0	0	17 (30.3)	6 (12)	4 (12.9)	19 (25.3)	6 (33.3)	17 (19.3)
Vancomycin	8 (7.54)	0	98 (92.45)	2 (3.8)	2 (40)	2 (4.6)	0	2 (50)	2 (3.5)	6 (12)	2 (6.4)	6 (8)	0	8 (9)
Clarithromycin	48 (45.28)	12 (11.32)	46 (43.39)	22 (42.3)	3 (60)	19 (44.1)	2 (50)	2 (50)	25 (44.6)	23 (46)	14 (45.1)	34 (45.3)	9 (50)	39 (44.3)
Erythromycin	72 (67.92)	14 (13.2)	20 (18.86)	34 (65.3)	3 (60)	31 (72)	2 (50)	2 (50)	38 (67.8)	34 (68)	27 (87)	45 (60)	14 (77.7)	58 (65.9)
Clindamycin	56 (52.83)	4 (3.77)	46 (43.39)	31 (59.6)	2 (40)	23 (53.4)	0	0	31 (55.3)	25 (50)	18 (58)	38 (50.6)	14 (77.7)	42 (47.7)
Azithromycin	48 (45.28)	24 (22.64)	34 (32.07)	26 (50)	2 (40)	20 (46.5)	0	0	26 (46.2)	22 (44)	14 (45.1)	34 (45.3)	4 (22.2)	44 (50)
Gentamicin	14 (13.2)	0	92 (86.79)	8 (15.3)	1 (10)	5 (11.6)	0	0	8 (14.2)	6 (12)	3 (9.6)	11 (14.6)	2 (11.1)	12 (13.6)
Kanamycin	61 (57.54)	0	45 (42.45)	30 (57.6)	1 (10)	26 (60.4)	2 (50)	2 (50)	29 (51.7)	32 (64)	19 (61.2)	42 (56)	6 (33.3)	55 (62.5)
Levofloxacin	72 (67.92)	6 (5.66)	28 (26.41)	36 (69.2)	1 (10)	31 (72)	2 (50)	2 (50)	39 (69.6)	33 (66)	21 (67.7)	51 (68)	16 (88.8)	56 (63.6)
Ofloxacin	83 (78.3)	0	23 (21.69)	40 (76.9)	3 (60)	36 (83.7)	2 (50)	2 (50)	43 (76.7)	40 (80)	26 (83.8)	57 (76)	15 (83.3)	68 (77.2)
Tetracycline	100 (94.33)	0	6 (5.66)	52 (100)	5 (100)	37 (86)	4 (100)	2 (50)	55 (98.2)	45 (90)	29 (93.5)	71 (94.6)	18 (100)	82 (93.1
Chloramphenicol	13 (12.26)	17 (16.03)	76 (71.69)	6 (11.5)	1 (10)	4 (9.3)	0	2 (50)	9 (16)	4 (8)	8 (25.8)	5 (6.6)	3 (16.6)	10 (11.3)
Ceftriaxone	56 (52.83)	0	50 (47.16)	32 (61.5)	2 (40)	22 (51.1)	0	0	33 (58.9)	23 (46)	14 (45.1)	42 (56)	10 (55.5)	46 (52.2)
Quinupristin-Dalfopristin	64 (60.37)	24 (22.64)	18 (16.98)	34 (65.3)	5 (100)	23 (53.4)	0	2 (50)	37 (66)	27 (54)	19 (61.2)	45 (60)	16 (88.8)	48 (54.5)
Linezolid	0	0	106 (100)	0	0	0	0	0	0	0	0	0	0	0

There was a significant relationship between the history of abortion and resistance to erythromycin (P = 0.02) and clindamycin (*P* = 0.002). Also, a significant relationship was observed between the number of abortions and resistance to penicillin (*P* = 0.007), clarithromycin (*P* = 0.004), clindamycin (*P* = 0.01), gentamicin (*P* = 0.001), and chloramphenicol (*P* = 0.003). On the other hand, a significant relationship was seen between the membrane rupture and resistance to azithromycin (*P* = 0.02), kanamycin (P = 0.02), and quinupristin-dalfopristin (P = 0.01).

Moreover, among the 30 isolates that were resistant or intermediate resistant to erythromycin but sensitive or intermediate resistant to clindamycin, 15 (50%) had a positive D-test. These isolates had the iMLS_B_ phenotype, and the remaining 15 (50%) isolates from this category belonged the M-phenotype group. Furthermore, 42 (39.62%) isolates had simultaneous resistance to erythromycin and clindamycin (cMLS_B_), and 7 (6.6%) isolates belonged to the L-phenotype group. Among 15 isolates with M-phenotype, 8 (61.53%), 8 (61.53%), and 8 (61.53%) were resistant to azithromycin, clarithromycin, and quinupristin/dalfopristin, respectively (Table 3). Also, among 15 isolates with iMLS_B_ phenotype, 12 (80%), 14 (93.33%), and 8 (53.33%) showed resistance to azithromycin, clarithromycin, and quinupristin/dalfopristin, respectively. On the other hand, among 42 isolates with cMLS_B_ phenotype, 25 (59.52%), 24 (57.14%), and 30 (71.42%) were resistant to azithromycin, clarithromycin, and quinupristin/dalfopristin, respectively. Moreover, among 7 isolates with L-phenotype, 2 (28.57%), 2 (28.57%), and 5 (71.42%) showed resistance to azithromycin, clarithromycin, and quinupristin/dalfopristin, respectively.

**Table 3:** The antimicrobial resistance pattern and the presence of resistance genes in different resistance phenotypes

Different phenotypes	No. (%) of isolates with different resistance pattern against									No. (%) of isolates containing		
	Azithromycin			Clarithromycin			Quinupristin-dalfopristin			*ermB*	*erm-TR*	*mefA/E*
	Resistant	Intermediate resistant	Susceptible	Resistant	Intermediate resistant	Susceptible	Resistant	Intermediate resistant	Susceptible			
cMLS_B_ (*n* = 42)	25 (59.52)	9 (21.42)	8 (19.04)	24 (57.14)	6 (14.28)	12 (28.57)	30 (71.42)	8 (19.04)	4 (9.52)	41 (97.61)	19 (45.23)	4 (9.52)
iMLS_B_ (*n* = 15)	12 (80)	1 (7.69)	2 (15.38)	14 (93.33)	1 (7.69)	0	8 (53.33)	5 (38.46)	2 (15.38)	15 (100)	2 (13.33)	0
M (n = 15)	8 (61.53)	5 (33.33)	2 (15.38)	8 (61.53)	1 (7.69)	6 (40)	8 (61.53)	5 (38.46)	2 (15.38)	15 (100)	6 (46.15)	2 (13.33)
L (n = 7)	2 (28.57)	1 (14.28)	4 (57.14)	2 (28.57)	2 (28.57)	3 (42.85)	5 (71.42)	0	2 (28.57)	7 (100)	3 (42.85)	2 (28.57)

### Resistance to antibiotics and the presence of antibiotic resistance genes

The *ermB* gene was the most prevalent resistance gene in the present study (Table 4). However, 100 (94.33%) *S. agalactiae* isolates contained this gene, while 71 were resistant to erythromycin. The *ermTR* gene was detected in 38 (35.84%) isolates, while 27 (71%) were resistant to erythromycin. The efflux encoding gene, *mefA/E*, had the lowest prevalence in this study, while 8 (7.54%) isolates contained this gene, and 4 (50%) were resistant to erythromycin. Moreover, *tetM* was the second prevalent resistance gene, while 79 (74.52%) isolates contained this gene, and all were resistant to tetracycline. Also, all 37 isolates containing the *tetO* gene were resistant to tetracycline. On the other hand, 20 (18.86%) isolates were *aphA3*-positive, however, 3 (15%) and 15 (75%) of them were gentamicin-high-level-resistant (GHLR) and amikacin-high-level-resistant (AHLR), respectively. However, other *aphA3*-positive isolates were susceptible to these aminoglycosides. The relationship between the presence of antibiotic resistance genes and resistance to different classes of antibiotics has been investigated statistically and listed in Table 4. Among the resistance genes, the *ermB* had a significant relation with resistance to erythromycin (*P* = 0.006) and azithromycin (*P* = 0.01). Also, a significant relation was observed between the presence of the *tetM* gene and resistance to tetracycline (*P* < 0.001). However, this gene was not present in any susceptible isolates, but was observed in more than 75% of the intermediate-resistant and resistant isolates.

**Table 4:** The relationship between the presence of antibiotic resistance genes and resistance to different antibiotics

Resistance genes	Antibiotics	No. (%) of isolates with different resistance pattern			*P*-*value*
		Resistant	Intermediate resistant	Susceptible	
*ermB* (*n* = 100)	Erythromycin	71 (71)	13 (13)	16 (16)	0.006
	Clarithromycin	47 (47)	11 (11)	42 (42)	0.34
	Azithromycin	48 (48)	23 (23)	29 (29)	0.01
	Clindamycin	54 (54)	4 (4)	42 (42)	0.47
*mefA/E* (n = 8)	Erythromycin	4 (50)	2 (25)	2 (25)	0.4
	Clarithromycin	6 (75)	0	2 (25)	0.1
	Azithromycin	6 (75)	2 (25)	0	0.1
	Clindamycin	6 (75)	0	2 (25)	0.4
*ermTR* (*n* = 38)	Erythromycin	27 (71.05)	6 (15.78)	5 (13.15)	0.49
	Clarithromycin	22 (57.89)	2 (5.26)	14 (36.84)	0.1
	Azithromycin	16 (42.1)	13 (34.21)	9 (23.68)	0.08
	Clindamycin	24 (63.15)	0	14 (36.84)	0.13
*TetO* (*n* = 37)	Tetracycline	37 (100)	–	0	0.05
*TetM* (*n* = 79)	Tetracycline	79 (100)	–	0	0.000
*aph-A3* (*n* = 20)	Gentamicin	3 (15)	–	17 (85)	0.79
	Kanamycin	15 (75)	–	5 (25)	0.08

### RAPD-PCR profiling of the isolates

**Fig. 1:**
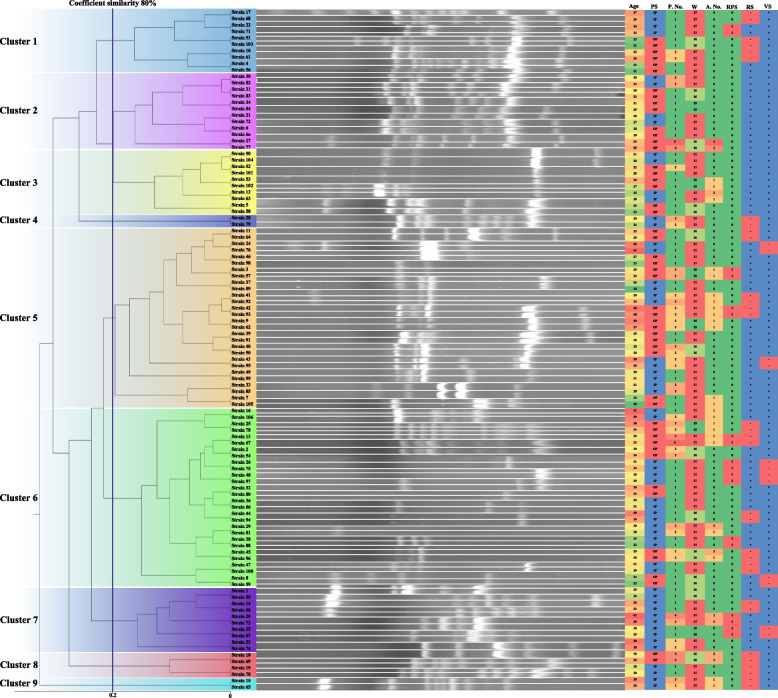
RAPD patterns of 106 *S. agalactiae* isolates collected from pregnant women in this study. The blots are cropped from the original electrophoresis gel and the blots with the same patterns are placed together. Also, the original gels with full-length blots are attached as supplementary files. Abbreviations: PS; patient status, P. no.; pregnancy number, W; week of pregnancy, A. no.; abortion number, RFS; rupture of fetal sac, RS; rectal swab, VS; vaginal swab, IP; inpatient, OP; outpatient. We cropped the gels from different parts of the same gel, and the cropped gels/blots are displayed in a supplementary information

All 106 *S. agalactiae* isolates obtained from pregnant women in this study were subjected to RAPD-PCR typing shown in Fig. 1. However, 1 to 11 DNA fragments with sizes of 180 bp to 4500 bp were obtained, while 53 different types were observed among the isolates. Also, nine various clones (clusters) of *S. agalactiae* were identified in this study with 80% similarity. We detected five groups with a 70% similarity in the present study. Four clusters (1–4) were detected in group 1 with a 70–80% similarity, while clusters 5 and 6 belonged to group 2, and the other three groups did not show any clustering. The highest number of isolates was related to clusters 5 and 6, each contained 28 isolates. However, the lowest number of isolates was observed in clusters 4 and 9, contained two isolates. Also, clusters 1, 2, 3, 7, and 8 contained 10, 12, 10, 10, and 4 isolates, respectively. Among the isolates of cluster 5 (*n* = 28), 14 (50%) isolates were obtained from hospitalized women, and 8 (28.57%) pregnant women had a history of abortion (once for each person), and four participants had a history of premature membrane rupture (once for each person). Also, among the isolates of cluster six, 16 and 12 were obtained from inpatients and outpatients. Also, 12 (42.85%) pregnant women had a history of abortion (two persons had twice and the rest once), and eight participants reported a history of premature membrane rupture. The highest percentage of abortion was observed in participants of clusters 8 and 6, and the highest premature membrane rupture was observed in participants of clusters 7 and 6. Also, the prevalence of antibiotic resistance genes in different clusters of *S. agalactiae* is shown in the Table 5.

**Table 5:** Prevalence of antibiotic resistance genes in different clusters of *S. agalactiae*

*S. agalactiae* clusters (No. of isolates)	The number (%) of isolates carrying antibiotic resistance genes					
	*ermB*	*ermTR*	*mefA/E*	*tetM*	*tetO*	*aphA3*
1 (n = 10)	8 (80)	2 (20)	0	6 (60)	2 (20)	2 (20)
2 (*n* = 12)	12 (100)	6 (50)	2 (16.66)	8 (66.66)	6 (50)	0
3 (n = 10)	8 (80)	4 (40)	0	6 (60)	4 (40)	4 (40)
4 (n = 2)	2 (100)	0	0	2 (100)	0	0
5 (n = 28)	28 (100)	12 (42.85)	2 (7.14)	22 (78.57)	14 (50)	4 (14.28)
6 (n = 28)	26 (92.85)	6 (21.42)	4 (14.28)	20 (71.42)	4 (14.28)	4 (14.28)
7 (n = 10)	8 (80)	4 (40)	0	10 (100)	8 (80)	2 (20)
8 (n = 4)	4 (100)	4 (100)	0	2 (50)	2 (50)	2 (50)
9 (n = 2)	2 (100)	0	0	2 (100)	0	2 (100)

## Discussion

*Streptococcus agalactiae* was first identified from the milk of cows with mastitis. However, this organism is currently involved as a pathogen causing invasive infections in infants and adults [24]. Group B Streptococci (GBS) can be isolated from the genitourinary and gastrointestinal tracts of about 25% of healthy adult women. However, 1% of infants born from these women are infected with GBS, while 10% die and a considerable percent of survived infants suffer from multiple neurological problems [25]. In our study, GBS was isolated and identified from 71 (16.9%) rectal and 95 (22.61%) vaginal samples. The rate of vaginal isolation in another study conducted in Brazil was higher than that the rectal samples [26]. The rates of GBS isolation from other studies from South Africa, China, Namibia, Belgium, and Ethiopia were 30.9%, 3.7–14.52, 5.7, 24, and 25.45%, respectively [7, 9, 19, 27].

Pregnant women and infants are considered as high-risk group for GBS infections. Formerly, penicillin was administered intravenously in mothers to prevent early infections caused by GBS [7, 16]. However, 21.69% of our isolates were resistant to penicillin, while the resistance rates to penicillin and ampicillin in *S. agalactiae* isolates collected from Ethiopian pregnant women (2016–2017) were 10.2 and 9.2%, respectively [13]. Besides, 100% of isolates collected in 2018 from 18 pregnant women in Namibia were susceptible to penicillin and ampicillin [19]. For mothers allergic to penicillin, erythromycin and clindamycin can use as second-line treatment [7, 16]. Meanwhile, resistance to these antibiotics is increasing, and empirical treatment is not recommend. Therefore, to choose the appropriate antibiotic therapy, it is necessary to perform an antibiotic susceptibility testing [21]. However, concomitant resistance of GBS isolates to erythromycin and clindamycin is a significant concern worldwide. The GBS resistance against erythromycin can be due to an efflux pump encoded by the *mefA/E* genes (M phenotype) or the methylation of the ribosomal 23 s rRNA mediated by the *erm* genes (MLS_B_ phenotype) [7]. In our study, 52.83 and 67.92% of the GBS isolates were resistant to clindamycin and erythromycin, respectively. These rates were higher than the results of another Iranian study [28]. In a study conducted by Castellano-Filho on 221 pregnant women in Brazil, 50 and 22.7% of the isolates were resistant to clindamycin and erythromycin [29]. However, the clindamycin resistance rate was consistent with the present study. The Spanish research conducted by Rojo-Bezares et al. in 2011 reported that 69.3% of their GBS isolates were cMLS_B_. However, 92.3% of these isolates contained the *ermB* gene, while 9.61% were *ermTR*-positive [30]. They reported that 98.7% of their cMLS_B_ isolates were resistant to azithromycin. Another study conducted in Ethiopia stated that 26.1% of their isolates were detected as cMLS_B_ [13]. However, we observed that 42 (39.62%) isolates were simultaneously resistant to erythromycin and clindamycin (cMLS_B_). Among these isolates, 41 (97.61%) contained the *ermB* gene. The relation of *ermB* gene with TN-*916* and TN-*1514* justifies the high prevalence of this gene in our isolates [16]. However, among our cMLS_B_ isolates, 59.52, 57.14, and 71.42% were resistant to azithromycin, clarithromycin, and quinupristin-dalfopristin, respectively. Moreover, the *ermB* gene is more prevalent in Asian countries, except for Hong Kong, where the *mefA/E* is more predominant in erythromycin-resistant GBS [16]. However, the *mefA/E* gene was detected in 4 (9.52%) cMLS_B_-positive GBS isolates of the present study. On the other hand, we found that 19 (45.23%) of these isolates contained the *ermTR* gene. Another study conducted by Heelan et al. in USA between 2002 and 2003 reported that just 12/200 (6%) GBS isolates collected from pregnant women showed a cMLSB phenotype. However, 8 (66.66%) isolates contained the *ermB* gene, 4 (33.33%) were *ermTR*-positive, while any of them were carried the *mefA* gene [31]. These differences indicate different antibiotic stewardship in various areas. As we detected that the rate of antibiotic-resistant isolates in the present study were almost equal in outpatients and inpatients, we may concluded that this high level resistance in our isolates is due to arbitrary use of antibiotics by people in our region. On the other hand, we detected that all iMLS_B_, M-phenotype, and L-phenotype isolates contained the *ermB* gene, while the *ermTR* gene was detected in 13.33, 46.15, and 42.85% of these isolates, respectively. However, Rojo-Bezares et al. in Spain reported that 62.5% of their iMLSB isolates were *ermTR*-positive, and all M-phenotype were *mefA*-positive [30]. Nevertheless, among our eight *mefA*-positive isolates, 4, 2, and 2 belonged to iMLSB, M-phenotype, and L-phenotype, respectively.

Also, tetracycline was the least effective antibiotic in the present study. The results of the present study showed that 94.33% of the isolates were resistant to tetracycline consistent to the study of Rojo-Bezares et al. [30]. This result was slightly more than studies conducted in Ethiopia [13], China [16], and USA [31]. However, Haimodi et al. reported that 100% of their isolates in Namibia were resistant to tetracycline [19]. On the other hand, we detected that among 100 tetracycline-resistant isolates, 79 and 37 contained the *tetM* and *tetO* genes, respectively, while none of the susceptible isolates carried these genes. However, 31 tetracycline-resistant isolates contained both *tetM* and *tetO* genes. Rojo-Bezares et al. reported that 67.6, 25, and 5.9% of their isolates were carrying *tetM*, *tetO*, and both of them, respectively [30]. These rates in China were 92, 5, and 1%, respectively [16], while in Namibia, 16/18 isolates were *tetM*-positive, and none contained the *tetO* gene [19]. Like *ermB*, *tetM* distribution in Streptococci is related to Tn-*916*-Tn-*1514* transposons [16], so higher prevalence of this gene in all studies is reasonable. However, this ratio is different in Hong Kong [16]. The high level tetracycline resistance and the high prevalence of *tetM* gene may be due to the shared use of tetracycline in humans and animals [19].

Although ribosomal mutations are the cause of resistance to aminoglycosides in vitro, most aminoglycoside-resistant clinical isolates are mediated by aminoglycoside-modifying enzymes [32]. Several enzymes can deactivate aminoglycosides, while APH-A3 is more problematic in *S. agalactiae* [16]. Considering that the *aph-A3* gene can transfer between bacteria through Tn-*916*-Tn-*1514* transposons [16], the presence of this gene can be a problematic concern in vaginal and rectal isolates of *S. agalactiae*. Another determinant for aminoglycoside resistance in GBS is a chromosome located Tn-*3706*, that can transpose onto the conjugative plasmid IP501 [33]. However, the *aph-A3* resistance gene was detected in 20 *S. agalactiae* isolates in the present study, while 3/14 (21.42%) and 15/61 (24.59%) were GHLR and AHLR, respectively. Other *aphA3*-positive isolates were susceptible to these aminoglycosides. This issue can be due to the presence of other aminoglycoside modifying enzymes or other resistance factors in the investigated strains. However, Granlund et al. reported that all of their kanamycin-resistant isolates contained the *aph-A3* gene [34]. Due to the synergistic bactericidal effect of aminoglycosides and penicillin [34], this combination may be effective in this area. However, the resistance rates against gentamicin and penicillin were 21.69 and 13.2%, respectively.

Besides, we detected 53 different RAPD types in our GBS isolates. However, nine various clones were clustered in the present study. Two broadest clones in this study each had 28 isolates, which 50% of them were isolated from hospitalized individuals. Also, 29 and 43% of the pregnant women categorized in clones 5 and 6 had a history of abortion. However, 14 and 28% had a history of premature membrane rupture. Besides, the most prevalence of abortion and amniotic sac rupture were observed in isolates belonged to cluster 6, indicating that our isolates had high variability. However, most isolates belonged to two clones exhibiting the distribution of these clones in all areas of Mazandaran province in north of Iran. On the other hand, almost an equal distribution of antibiotic resistance genes was observed in different clones of the present study, indicating the same sources of the isolates. However, Martinez et al. from Canada were observed five groups among 38 isolates collected from pregnant women using three primers, while three groups did not present any clustering concordant with the present study [23].

## Conclusion

This study showed a high rate of antibiotic resistance in *S. agalactiae*, as a vaginal and rectal normal flora, while some of this antibiotics, such as quinopristin-dalfopristin, are not used in the clinical settings of Iran. However, 60.37% of our isolates were resistant to this antibiotic. The high-level resistance may be due to the arbitrarily use or the use of some antibiotics in the livestock industry. Resistance to macrolides, tetracycline, fluoroquinolones, and quinupristin/dalfopristin is a significant concern in the community. These results and the high presence of transferable genes, *ermB* and *tetM*, in isolates collected from pregnant women indicate the importance of antibiotic management in this area. One of the most important reasons for the increase of these antibiotic resistance rates may be the preventive use of some of them in pregnant women. Thus, the need to re-considering this problem seems to be necessary.

## Supplementary Information

**Additional file 1.**

## Data Availability

All data generated or analyzed during this study are included in this published article.

## Abbreviations

DNA: Deoxy ribonucleic acid
RAPD: Randomly amplified polymorphic DNA
MLS_B_ resistance: Macrolide, lincosamide, streptogramin B resistance
iMLS_B_: Inducible macrolide, lincosamide, streptogramin B resistance
cMLS_B_: Constitutive macrolide, lincosamide, streptogramin B resistance
GBS: Group B streptococcus
EOD: Early-onset disease
LOD: Late-onset disease
RPPs: Ribosomal protective proteins
CAMP: Christie, Atkins, Munch, and Peterson
ATCC: American type culture collection
CLSI: Clinical and laboratory standards institute
ICR: Inducible clindamycin resistance
PCR: Polymerase chain reaction
