# Supplementary figures and images for: Antibiotic resistance genes and molecular typing of Streptococcus agalactiae isolated from pregnant women
Source: BMC Pregnancy Childbirth. 2023 Jan 19;23:43. doi: 10.1186/s12884-023-05380-4 (PMC9854082; doi:10.1186/s12884-023-05380-4)

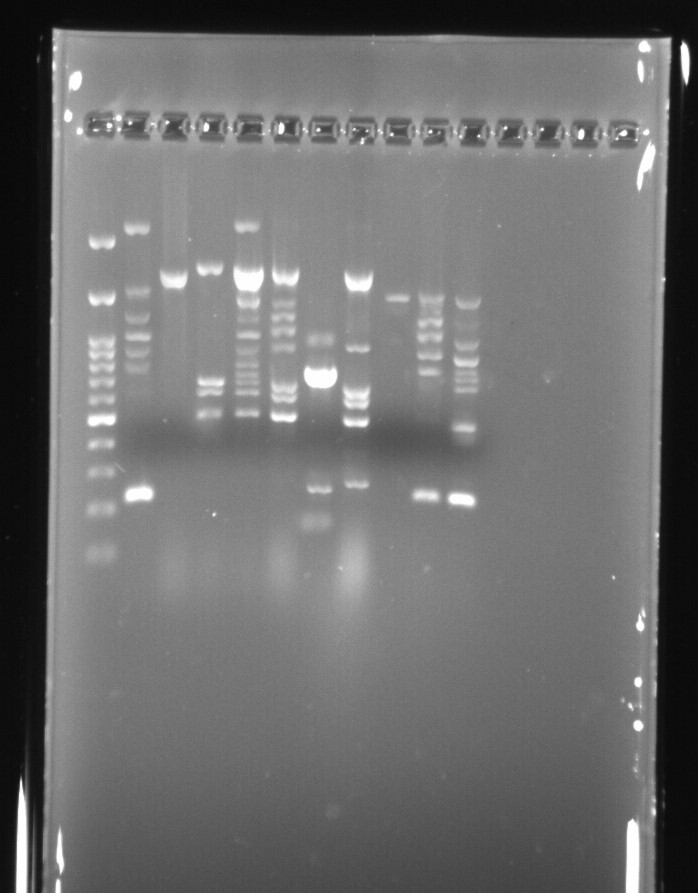

Supplement: Supplementary file 1 — Additional file 1. [file 12884_2023_5380_MOESM1_ESM.zip › 1,2,3,4,5,11,12,13,14,15.jpeg]

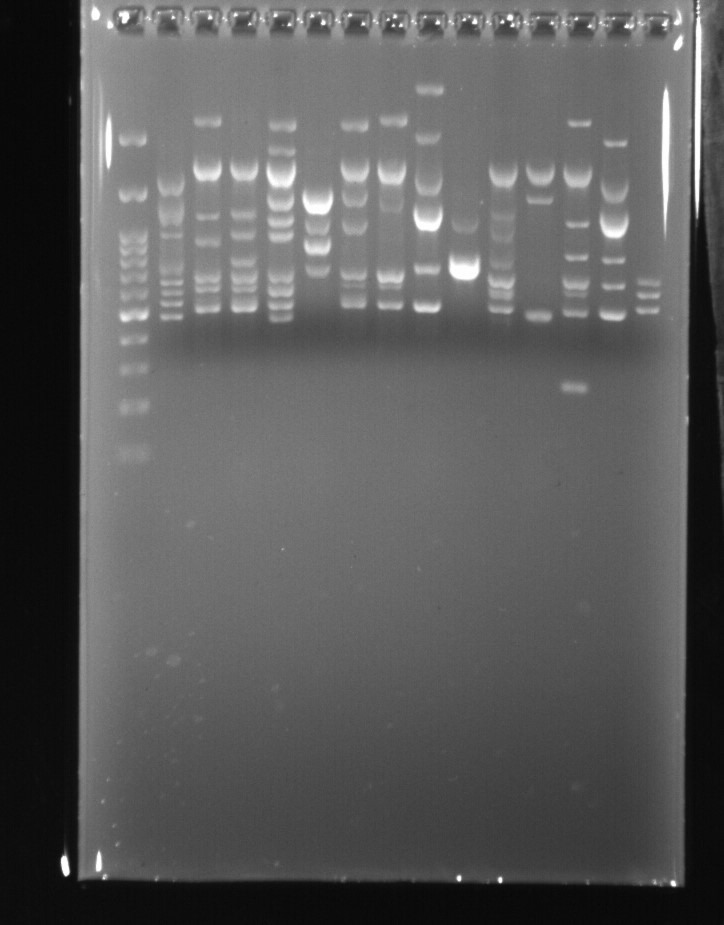

Supplement: Supplementary file 1 — Additional file 1. [file 12884_2023_5380_MOESM1_ESM.zip › 16-29.jpeg]

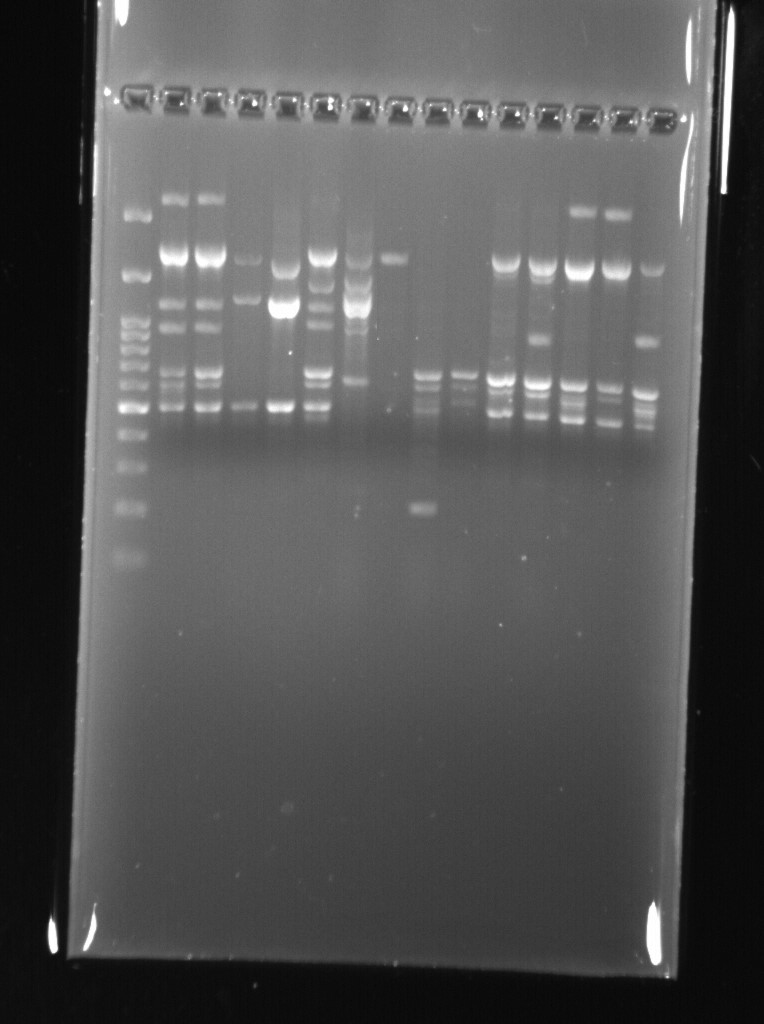

Supplement: Supplementary file 1 — Additional file 1. [file 12884_2023_5380_MOESM1_ESM.zip › 30-43.jpeg]

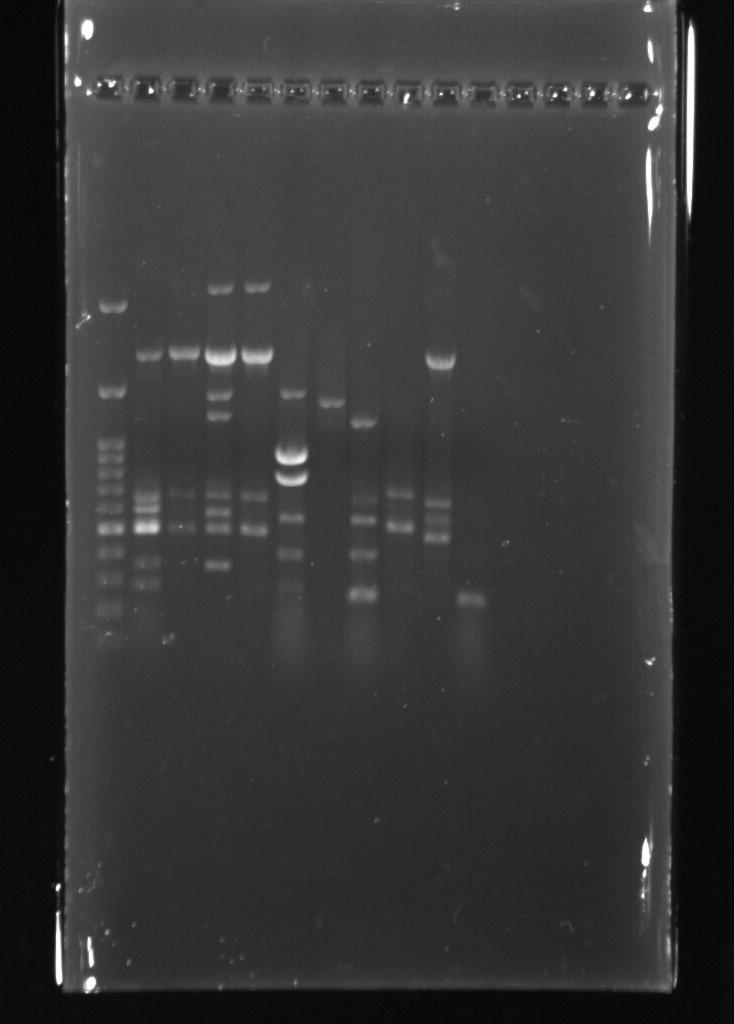

Supplement: Supplementary file 1 — Additional file 1. [file 12884_2023_5380_MOESM1_ESM.zip › 44-53 (right to left).jpeg]

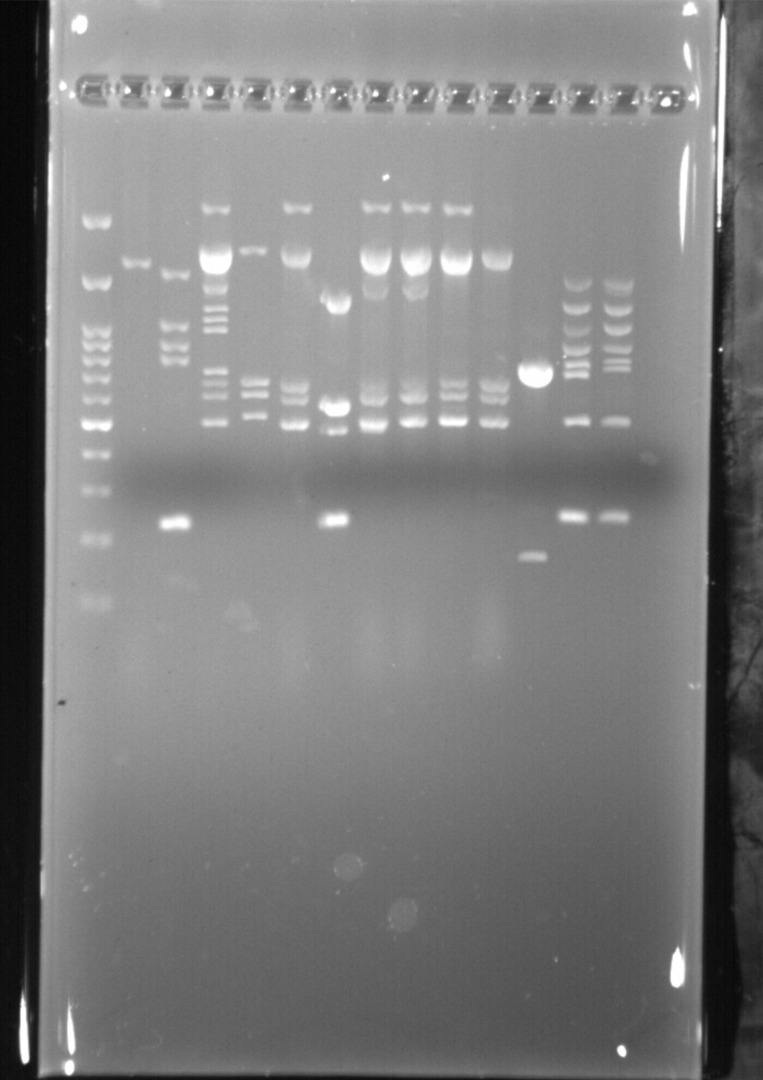

Supplement: Supplementary file 1 — Additional file 1. [file 12884_2023_5380_MOESM1_ESM.zip › 54-67.jpeg]

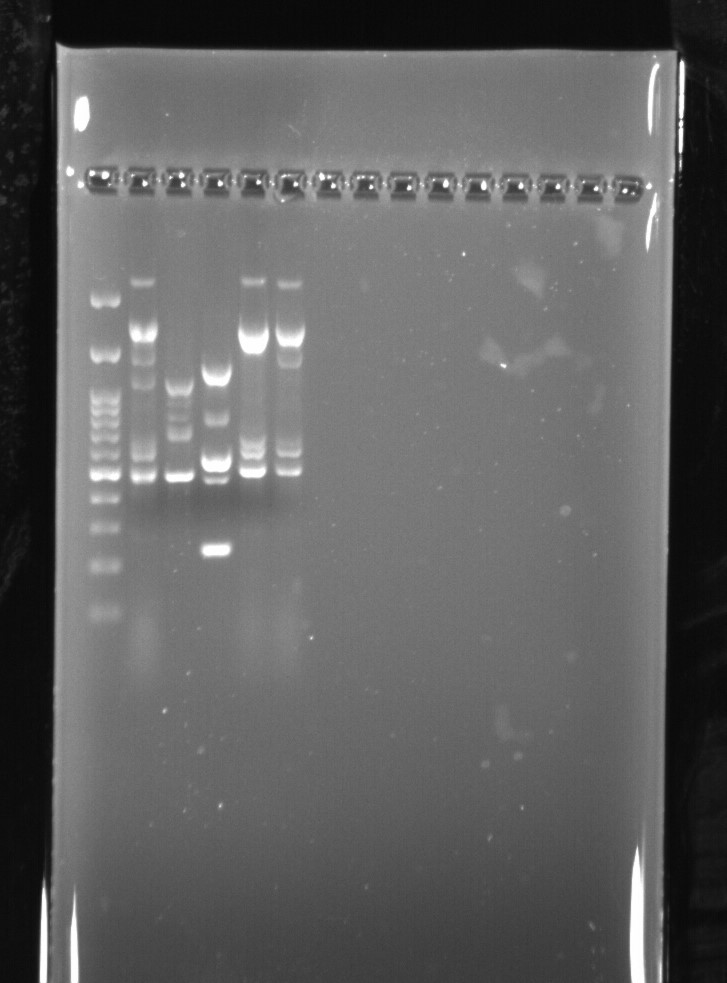

Supplement: Supplementary file 1 — Additional file 1. [file 12884_2023_5380_MOESM1_ESM.zip › 6-10.jpeg]

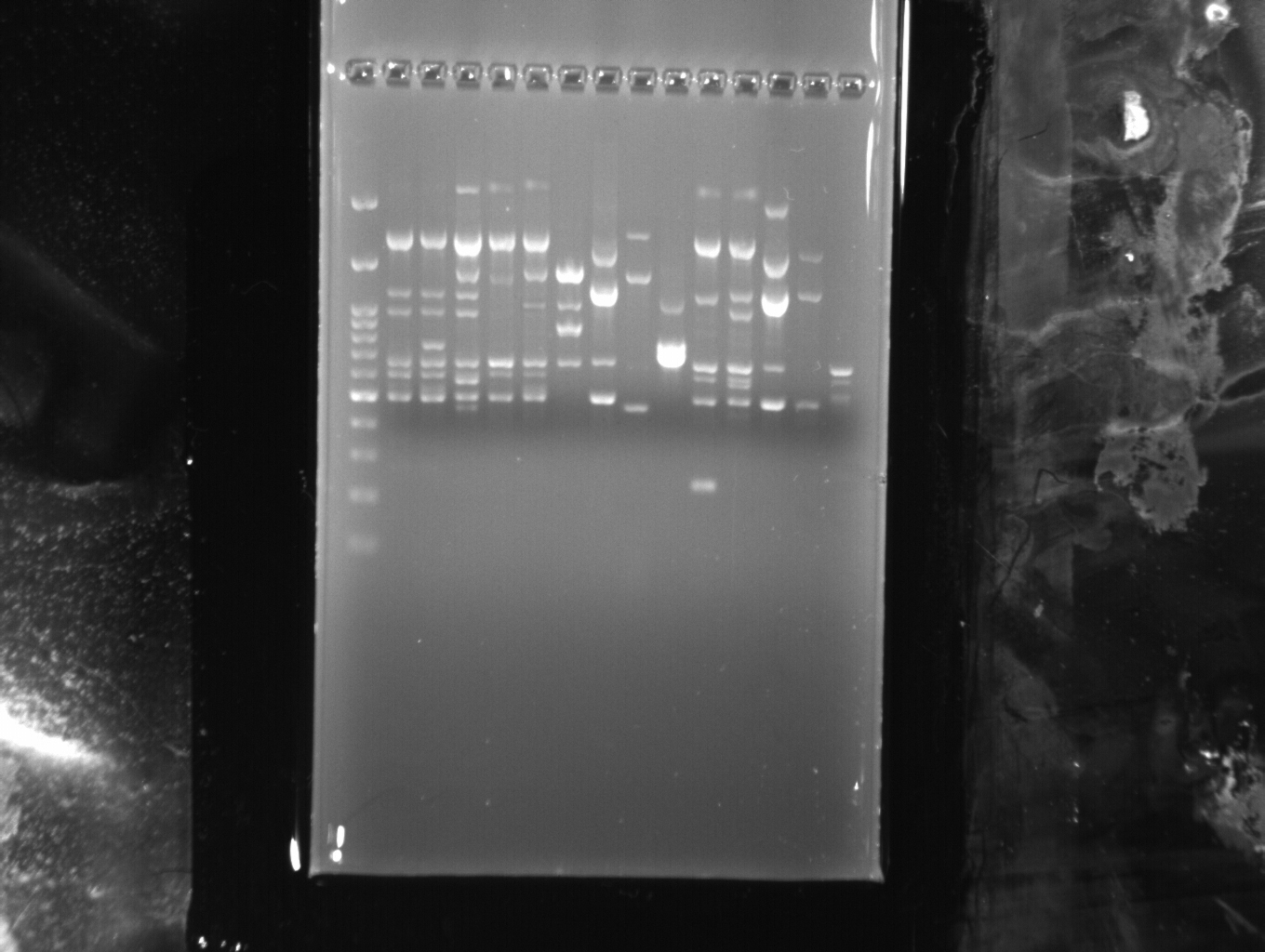

Supplement: Supplementary file 1 — Additional file 1. [file 12884_2023_5380_MOESM1_ESM.zip › 68-81.Jpg]

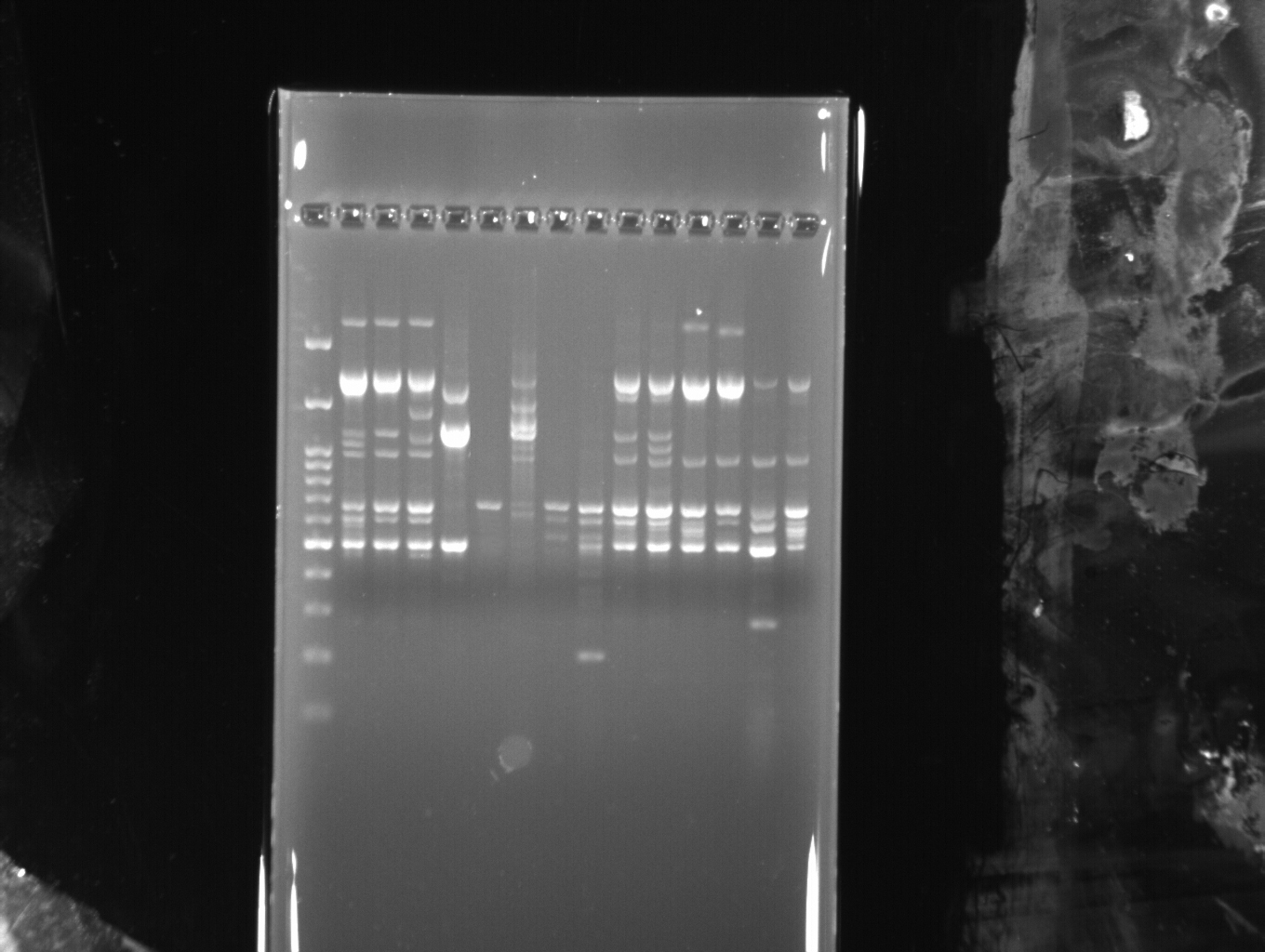

Supplement: Supplementary file 1 — Additional file 1. [file 12884_2023_5380_MOESM1_ESM.zip › 82-95.Jpg]

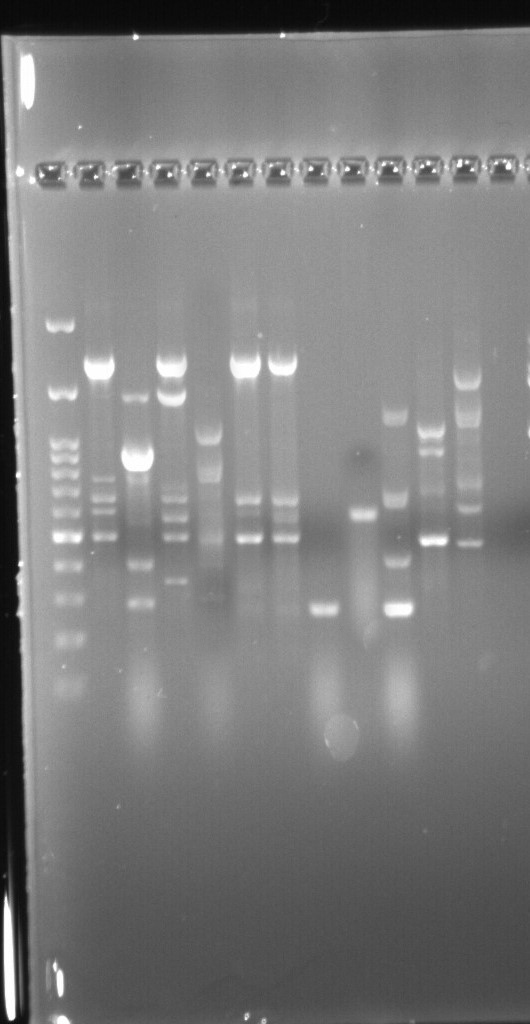

Supplement: Supplementary file 1 — Additional file 1. [file 12884_2023_5380_MOESM1_ESM.zip › 96-106.jpg]

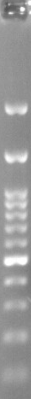

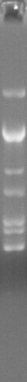

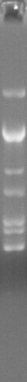

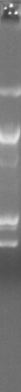

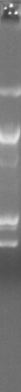

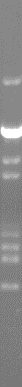

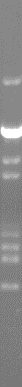

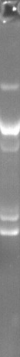

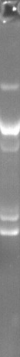

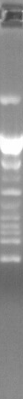

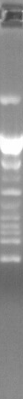

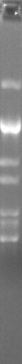

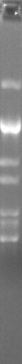

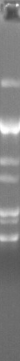

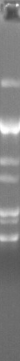

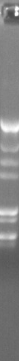

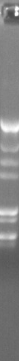

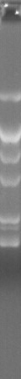

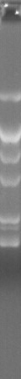

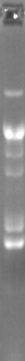

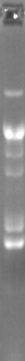

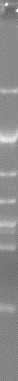

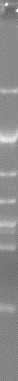

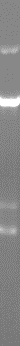

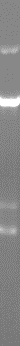

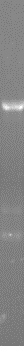

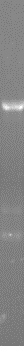

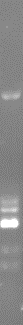

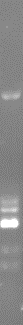

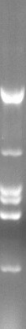

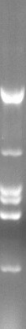

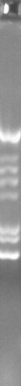

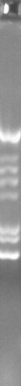

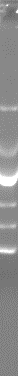

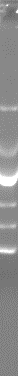

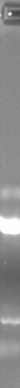

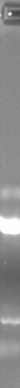

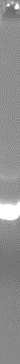

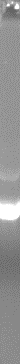

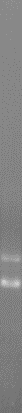

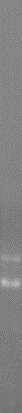

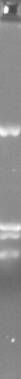

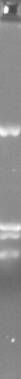

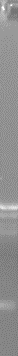

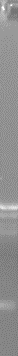

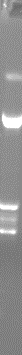

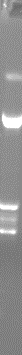

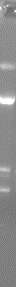

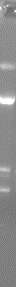

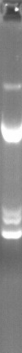

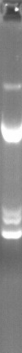

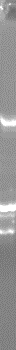

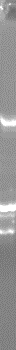

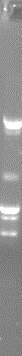

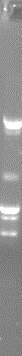

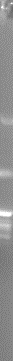

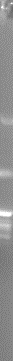

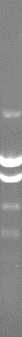

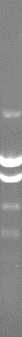

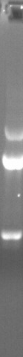

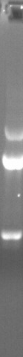

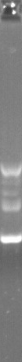

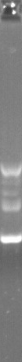

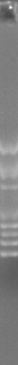

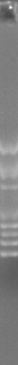

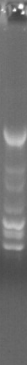

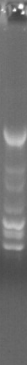

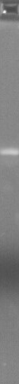

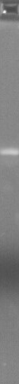

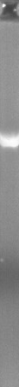

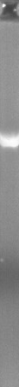

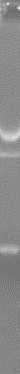

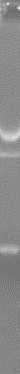

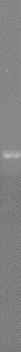

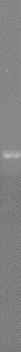

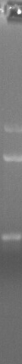

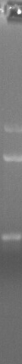

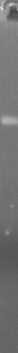

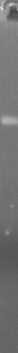

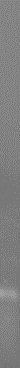

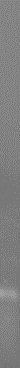

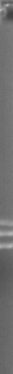

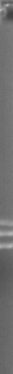

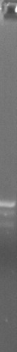

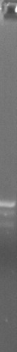

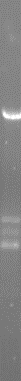

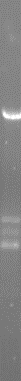

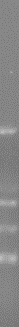

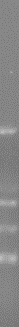

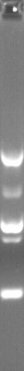

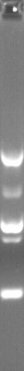

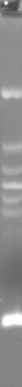

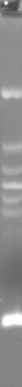

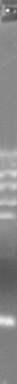

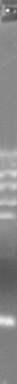

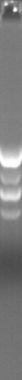

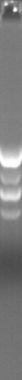

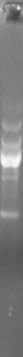

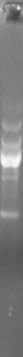

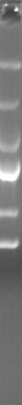

Supplement: Supplementary file 1 — Additional file 1. [file 12884_2023_5380_MOESM1_ESM.zip › Supplementary Information.docx]
